# Background subtraction for night videos

**DOI:** 10.7717/peerj-cs.592

**Published:** 2021-06-10

**Authors:** Hongpeng Pan, Guofeng Zhu, Chengbin Peng, Qing Xiao

**Affiliations:** 1 College of Information Science and Engineering, Ningbo University, Ningbo, China; 2 Ningbo Institute of Industrial Technology, Chinese Academy of Sciences, Ningbo, China, Ningbo, Zhejiang, China; 3 Electrical Engineering and Computer Science, Leibniz University Hannover, Hanover, Germany

**Keywords:** Background Subtraction, Night Videos

## Abstract

Motion analysis is important in video surveillance systems and background subtraction is useful for moving object detection in such systems. However, most of the existing background subtraction methods do not work well for surveillance systems in the evening because objects are usually dark and reflected light is usually strong. To resolve these issues, we propose a framework that utilizes a Weber contrast descriptor, a texture feature extractor, and a light detection unit, to extract the features of foreground objects. We propose a local pattern enhancement method. For the light detection unit, our method utilizes the finding that lighted areas in the evening usually have a low saturation in hue-saturation-value and hue-saturation-lightness color spaces. Finally, we update the background model and the foreground objects in the framework. This approach is able to improve foreground object detection in night videos, which do not need a large data set for pre-training.

## Introduction

Background subtraction aims to identify moving objects from current video frames with the knowledge of a background model (Sobral & Vacavant, 2014). It is a very useful image preprocessing tool in many applications. For example, in video surveillance, background subtraction can improve object tracking and recognition (Kim & Jung, 2017). General background subtraction models consist of three parts: background initialization builds the initial background model from a few frames at the beginning. Foreground detection extracts moving objects from the current frame by comparing with the background model, while background maintenance updates the background model (Bouwmans, 2012).

Background subtraction has been of interest for to researchers for decades, and most detection algorithms have used the pixel-based approaches. Some researchers consider background pixel values at each location of video frames follow a Gaussian distribution, and others propose the use of the median value of each location as the corresponding background pixel (Piccardi, 2004). Some researchers take the pixel color frequency into account using weightless neural networks in an unsupervised mode (De Gregorio & Giordano, 2017). For video surveillance systems, Gaussian mixture models have been used to cluster foreground and background pixels, respectively (Goyal & Singhai, 2017). Van Droogenbroeck & Paquot (2012) use a number of post-processing methods, such as eliminating small foreground blobs, to improve the performance. Their work is based on classifying foreground and background pixels and updating the background models accordingly. Probabilistic approaches (Ren, Zhang & Zhang, 2019) along with principle component analysis has also been used (Umer et al., 2021).

To improve the performance, researchers also propose to use descriptors. Some researchers transform the pixels from RGB space into other color spaces to separate color intensity from other color information (Balcilar, Amasyali & Sonmez, 2014; Martins et al., 2017). Another effective descriptor for background subtraction is texture-based local binary pattern (LBP) (Heikkila & Pietikainen, 2006). This descriptor combines neighboring pixels rather than single color information, and can better represent the local information of objects. Local binary similarity pattern (LBSP) is an improved LBP that uses larger patterns. St-Charles, Bilodeau & Bergevin (2015) used spatial information and temporal difference as an LBSP descriptor. Tan & Triggs (2010) developed local ternary patterns (LTP) which categorizes pixels into three threshold values.

Monitoring at night is also important for surveillance systems. Some researchers propose to use contrast analysis to capture local change over time to detect potential objects and then use spatial nearest neighbors to suppress false alarms (Huang et al., 2008). Some others use support vector machines and a combination of Kalman filter for object tracking (Fengliang Xu, Xia Liu & Fujimura, 2005). With a high performance background subtraction algorithm, such object detection algorithms can better focus on the moving objects.

However, most of the background subtraction these approaches are not designed for surveillance systems at night, because dark objects and reflection lights can significantly impact the performance of segmentation algorithms. In this paper, we propose a framework that utilizes multiple feature extractors to improve performance. Our contributions are:

1. We propose an unsupervised framework for background segmentation.
2. We propose a local pattern enhancement method for texture feature extraction in the evening.
3. We propose a combination of feature extractors to effectively obtain foreground objects in night videos.

## Related Works

Most state-of-art algorithms such as SUBSENSE (St-Charles, Bilodeau & Bergevin, 2015), WeSamBE (Jiang & Lu, 2018) and C-EFIC (Allebosch et al., 2015) use the pixel-wise RGB descriptors, which are effective for describing the change of objects. Such algorithms have a similar ability to consider the absolute difference of color intensity between the current frame and the background model only. Unfortunately, the RGB descriptor is sensitive and may mistakenly classify the foreground. For example, strong lighting in night videos can become false positive foreground objects.

Researchers have proposed local patterns, including Local Binary Pattern(LBP) (Heikkila & Pietikainen, 2006), Local Ternary Pattern(LTP) (Tan & Triggs, 2010) and Local Binary Similarity Pattern (LBSP) (Bilodeau, Jodoin & Saunier, 2013) to obtain local textural information. One common characteristic of those patterns is that the local difference information is given as ±1 or 0 according to the threshold *T*_*h*_.

Many model updating mechanisms have been proposed to date. Wang & Suter (2007) introduced the idea that the samples will be replaced according to its lifespan. Another effective solution (Barnich & Van Droogenbroeck, 2011) is using a stochastic strategy to update background samples. Both of these strategies are lacking evidence to decide whether current selected images should be replaced. An alternative solution was introduced by Jiang & Lu (2018) depends on the weight of each image.

We propose a framework that utilizes and develops multiple feature extractors and can significantly improve the performance of background subtraction in night videos.

## Method

**Figure 1:**
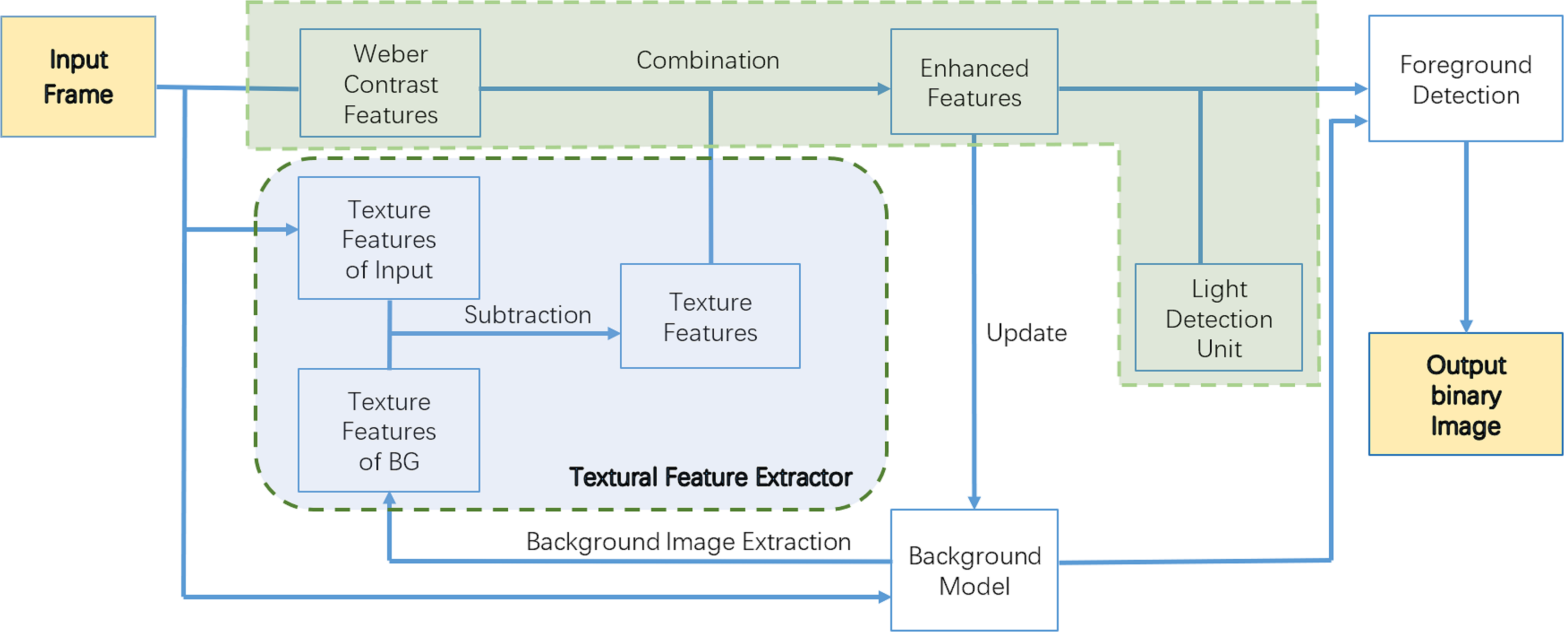
Framework of WCLPE.

The framework of our proposed approach is shown in Fig. 1 and can be divided into four parts, namely: the Weber contrast descriptor, an enhanced local feature extractor, a light detection unit, and a background model.

 Firstly, we define a background model. Video frames are captured and some of them are stored as “samples” in the pixel-level model, and these samples as a whole form our background model. We store a maximum of twenty-five samples in our background model. Secondly, we extract Weber features and texture features of input images, and compare with images in the background models, to obtain an enhanced representation of texture features through background subtraction. Finally, after eliminating the influence of illumination through the light unit detection, we separate the foreground objects through the foreground detection and output the results.

### Weber contrast descriptor

**Figure 2:**
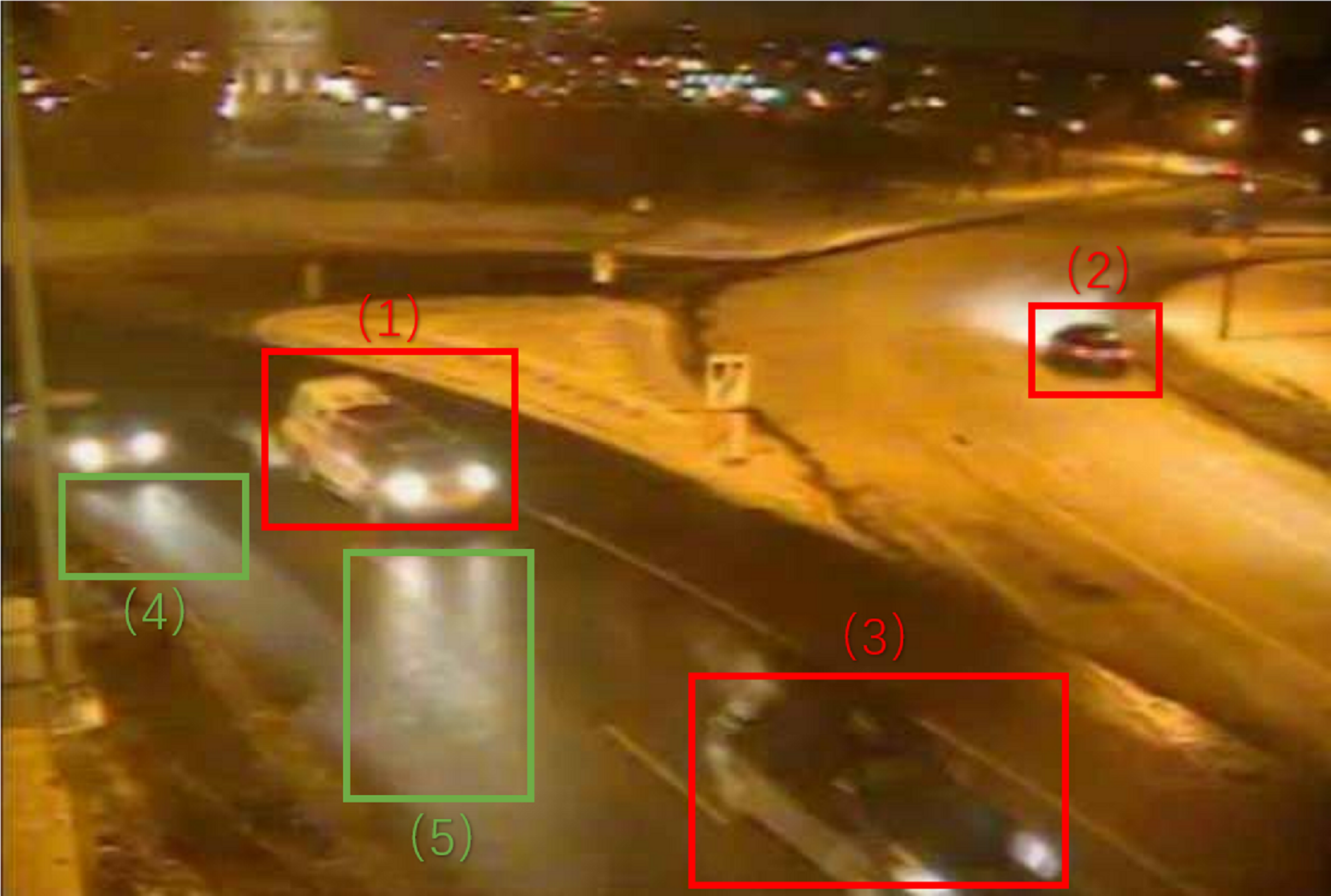
Winter Street #001225 (1), (2), (3): dim foreground object. (4), (5): strong lighted area.

As an example, in Fig. 2, area (3) contains a dim foreground object which has a small color deviation from the background samples, and the threshold to distinguish it from background should be small. In area (2), the pixel changes in the foreground object is much greater, so the threshold should be larger.

**Figure 3:**
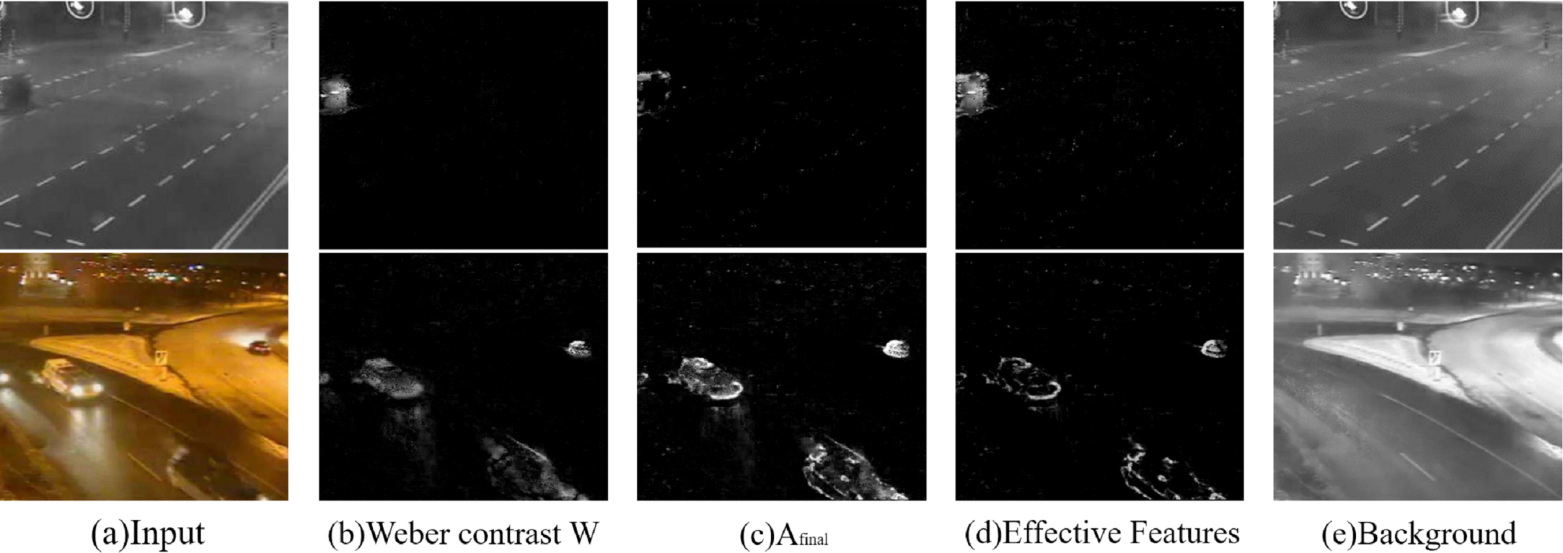
Feature maps. Input images: streetCornerAtNight-#002834 and winterStreet-#001225 (Wang et al., 2014).

To address this problem, we propose a more effective pixel-wise descriptor named the Weber contrast descriptor, which is a term borrowed from the Weber-Fechner law (Fechner, 1860; Fechner, 1966). This law indicates that visual systems have different sensitivity to changes in lightness when lightening is different (Fechner, 1860; Fechner, 1966). For example, in dim areas, our visual perception system can catch subtle change but in bright areas, it is more difficult to perceive same changes. With this idea, we define our Weber contrast descriptor in a simplified version as follows:

(1) \begin{eqnarray*}W= \frac{\Delta I}{I} ,\end{eqnarray*}

where Δ*I* is the actual change, namely, the deviation of intensity between background and current frame, and *I* is intensity of current frame. Equation (1) shows that objects in lighted area have relatively low Weber contrast values while objects in dim areas have relatively high values. Therefore, the detection of dim foreground objects is more effective, and the impact of lighting is not too great with the improved descriptor. The Weber contrast features are shown in Fig. 3B.

### Textural features

#### Local pattern enhancement

The Weber contrast descriptor requires the elimination of strong lighting influences as well as another light detection method. Hence, we propose another light detection mechanism for further elimination of light.

A lighted area gradually fades in background with a smooth decrease of intensity. On the contrary, the foreground region of interests (FROI) like cars and pedestrians, have obvious silhouettes in common, which means that we can distinguish FROI and lights from changing areas using this edge features.

**Figure 4:**
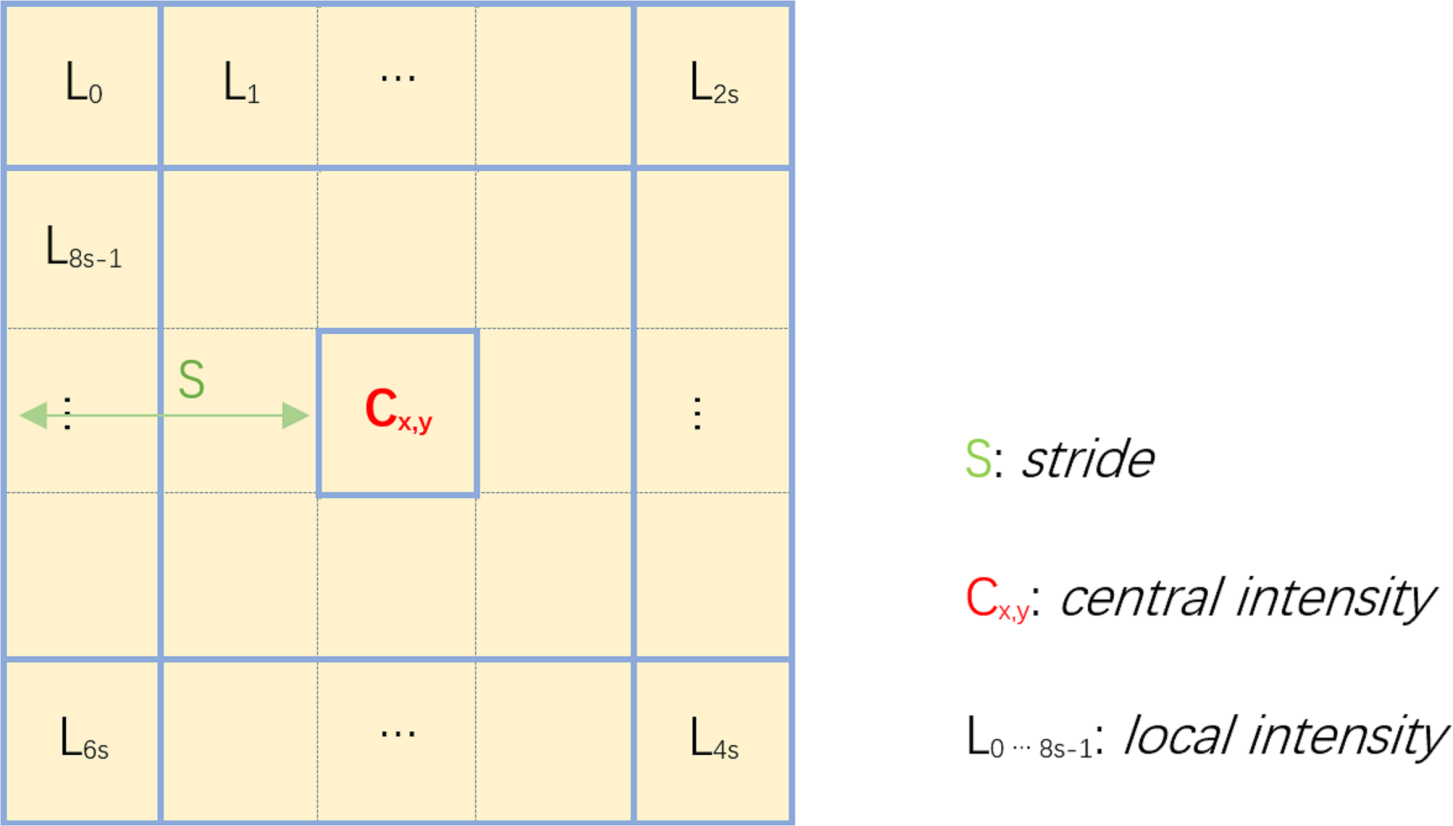
Local pattern enhancement.

We seek to obtain more local textual information to better describe the silhouette of FROI. Inspired by local binary patterns, we design a local pattern that includes more detailed information, as shown in Fig. 4 to resolve the problem. With this pattern, we consider the differences between all the marginal pixels and the center pixel, and then summarize the differences. We use the summation as a new descriptor, to indicate local textural features at pixel *x*, *y*. Mathematically, it is defined as ${\mathop{\sum }\nolimits }_{i=0}^{8s-1}{|}{L}_{i}-{C}_{x,y}{|}$, where *s* is the stride of pattern we have used in Fig. 4. In order to satisfy the condition to detect dim objects without strong lighting, the calculation of *A*(*x*, *y*) is modified as:

(2) \begin{eqnarray*}A(x,y)=\sum _{i=0}^{8s-1}{|}{L}_{i}-{C}_{x,y}{|} \ast \frac{{I}_{d}}{max({C}_{x,y},{T}_{c})} ,\end{eqnarray*}

where *I*_*d*_ is intensity degree, meaning that when the brightness is less than *I*_*d*_, the textural features should be enhanced. Otherwise, in high color intensity areas, the textural features will be suppressed by the multiplication of a small coefficient. *T*_*c*_ corresponds to threshold of camouflage. In low intensity areas, *A*(*x*, *y*) is very large because of the additional term, so we set the threshold *T*_*c*_ at 75 by default to avoid such large value. We need to normalize *A*(*x*, *y*) to combine the textural features with the Weber contrast features, which is shown in the following equation:

(3) \begin{eqnarray*}{A}_{norm}(x,y)= \frac{A(x,y)}{255} ,\end{eqnarray*}

where *s* is stride with default value two. This is the Local Pattern Enhancement (LPE) approach.

#### Textural feature extraction

The local binary features based on LBP or LBSP can be stored in one or two bytes. Similarly, the LTP pattern can be separated into upper LBP and lower LBP meaning that each image could include color information and textural features together without utilizing a large amount of memory. In order to maintain more information in the feature, we consider extracting the background image from background model, and extracting the local textural features from the background image and the current frame. Samples with the most similar are stored in the background model. To better represent the background, each pixel of the background image was taken from the average value of the corresponding pixels from *N* background samples (*N* = 30), as shown shown in Fig. 3E. Finally, to reduce the noise, the calculation of the final utilized textural feature *A*_*final*_ is based on the textural feature difference of the current frame and the background model, as below:

(4) \begin{eqnarray*}\begin{array}{@{}ll@{}} \displaystyle {A}_{final}(x,y)&\displaystyle =min\{\sum _{i=-1}^{1}\sum _{j=-1}^{1}max[0,{A}_{norm}^{cur}(x,y)-{A}_{norm}^{bg}(x+i,y+j)]\} \end{array}\end{eqnarray*}

where ${A}_{norm}^{cur}$ and ${A}_{norm}^{bg}$ are the normalized amplitudes of the current frame and the background image, while *x* and *y* are the positions in the images. This rule assumes that the background image is smoother than the current frame. The final utilized textural feature maps are shown in Fig. 3C.

### Light detection unit

We introduce detection of the light in night videos by combining an intrinsic attribute of color and saturation. There are many alternative representations of the RGB color model according to different applications, HSV (Hue-Saturation-Value) or HSL (Hue-Saturation-Lightness) (Joblove & Greenberg, 1978). Color space is one of such representations. The lighted areas in night videos are similar to natural light, which has a low saturation. Thus, the lighted areas can be separated from other areas in HSV/HSL color space by using a specific threshold on saturation.

**Figure 5:**
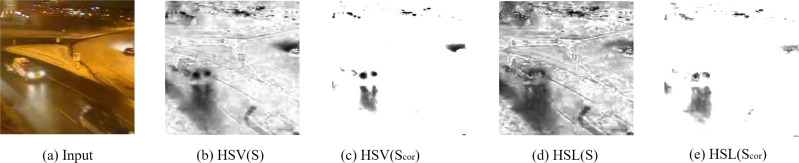
Saturation maps. Saturation maps. Input image: winterStreet-#001225 (Wang et al., 2014).

In our case we only consider the terms saturation (S) and value (V). The definition of saturation in HSV and HSL is only slightly different in that the colors with maximal saturation locate at lightness 0.5 in HSL, while they locate at value 1 in HSV. Light sources typically have low saturation but high value in HSV, such as headlights belong to foreground objects. Coincidentally, in HSL they have a relative high saturation just like other non-lighting objects. Thus, HSL is more suitable for our approach. Figure 5 shows the saturation maps calculated in HSV and HSL color space.

White or almost-gray objects have low saturation and the direct use of saturation will cause more false negatives. According to the Dark Channel Prior (He, Sun & Tang, 2011), in normal objects, there is at least one low intensity channel, but in lighted objects, characteristic are more like sky patches. Therefore, we can improve the saturation *S*_*cor*_ as follows:

(5) \begin{eqnarray*}{S}_{cor}(x,y)= \left\{ \begin{array}{@{}rcl@{}} \displaystyle &\displaystyle 1&\displaystyle \text{if} \frac{maxRGB(x,y)-minRGB(x,y)}{{N}_{all}} \lt {T}_{d}\text{}\\ \displaystyle &\displaystyle \frac{S(x,y)}{minRGB(x,y)} &\displaystyle \text{otherwise} \end{array} \right. \end{eqnarray*}

where *S* represents saturation in HSL, *N*_*all*_ is the number of pixels of an input image, and *T*_*d*_ is an upper boundary, *minRGB*(*x*, *y*) and *maxRGB*(*x*, *y*) are the minimum value and the maximum value of three channels (R, G, and B) for pixel position (*x*, *y*). In HSL biconical space, the closer the color vector is to the axis, the smaller saturation it has, and the saturation corresponding to the gray color is zero. Therefore, the light detection unit works only when the RGB information is not close to the gray value. When images taken at night are very close to gray images, the saturation method is not useful and we set *S*_*cor*_(*x*, *y*) = 1 in this case.

### Background match and classification

We have introduced the necessary features in our approach. The matching and background classification strategy are also important and must be combined to work effectively. One of the most important combined features in Fig. 1 is the effective feature which was calculated by following equation:

(6) \begin{eqnarray*}{F}_{effective}=W\ast {S}_{cor}+{A}_{final}^{2},\end{eqnarray*}

where *W*, *S*_*cor*_ and *A*_*final*_ are the matrices corresponding to Weber contrast, corrected saturation and final utilized textural features respectively. The value of each pixel is at the corresponding position in the matrix. In fact, the lighted areas masked by light detection units belong to other motion objects and were not classified as FROI. Thus, their pixels should not be updated into the background model, and the model update must be separated from the classification. Here the additional enhanced features *F*_*enhanced*_ for model update was defined as:

(7) \begin{eqnarray*}{F}_{enhanced}=W+{A}_{final}^{2}.\end{eqnarray*}

The process of classification is similar to SuBSENSE (St-Charles, Bilodeau & Bergevin, 2015), this process is presented in Eq. (8),

(8) \begin{eqnarray*}{S}_{mask}= \left\{ \begin{array}{@{}rcl@{}} \displaystyle 1&\displaystyle &\displaystyle \text{if}#\{{F}_{effective,i}\lt R,\forall i\}\lt #min\text{}\\ \displaystyle 0&\displaystyle &\displaystyle \text{otherwise} \end{array} \right. \end{eqnarray*}

(9) \begin{eqnarray*}{M}_{mask}= \left\{ \begin{array}{@{}rcl@{}} \displaystyle 1&\displaystyle &\displaystyle \text{if}#\{{F}_{enhanced,i}\lt R,\forall i\}\lt #min\text{}\\ \displaystyle 0&\displaystyle &\displaystyle \text{otherwise} \end{array} \right. \end{eqnarray*}

where *S*_*mask*_ is a segment mask for background segmentation while *M*_*mask*_ is a motion mask for model update. *R* is the matching threshold. The symbol #{.} means the number of true elements. #*min* is defined as the minimum number of matches, and the parameter #*min* = 2 is also used.

### Background Model Update

To obtain a dynamic background model, once the pixel of the current detected frame is close to the background samples, this pixel should be updated into the background model. At the same time, the image with the farthest distribution in the background model should be replaced. We set a weight for each image to decide which image should be replaced or not. The similar images are allocated by higher weights, and vice versa. The definition of the weight for samples is as following equations:

(10) \begin{eqnarray*}{S}_{mask}= \left\{ \begin{array}{@{}rcl@{}} \displaystyle {W}_{i}+2&\displaystyle &\displaystyle \text{if}{|}{I}_{i,s}-{I}_{cur}{|}\lt {T}_{lower}\text{}\\ \displaystyle {W}_{i}+1&\displaystyle &\displaystyle \text{if}{|}{I}_{i,s}-{I}_{cur}{|}\lt {T}_{upper}\text{}\\ \displaystyle {W}_{i}-1&\displaystyle &\displaystyle \text{otherwise} \end{array} \right. \end{eqnarray*}

where *I*_*i*,*s*_ is intensity of the *i*th image, while *I*_*cur*_ is the intensity of current frame. *T*_*upper*_ corresponds to threshold *R*_*color*_ in Jiang & Lu (2018) whose value is 23, while *T*_*lower*_ was given as 10 to increase the weight of highly similar images. Such updating strategy can ensure the high correlation of inner images. The lowest weight of image was replaced.

Additionally, to eliminate ghost objects, we adopt the policy of random updating neighboring pixels with time subsampling (St-Charles, Bilodeau & Bergevin, 2015). It should be noted that the updating policy of update is only suitable for neighboring pixels, and update of the current pixel is triggered when *M*_*mask*_ = 1.

## Result

Our approach has been evaluated based on CDnet2014 (Wang et al., 2014) to compare it with other algorithms and obtain an objective evaluation. This dataset incorporates 11 categories with a variety of scenes, including challenging weather, shadow, and night videos. We used night videos, including six different videos, namely tramStation, fluidHighway, brigeEntry, busyBoulvard, streetCornerAtnight, and winterStreet.

### Parameter Initialization

We initialized a few parameters, and the threshold of the Weber contrast was one of the most important. The Weber contrast descriptor is highly sensitive in dark areas, while in some scenes such dark objects belong to FROI but in other scenes are noises such as shadows. Thus, a proper strategy to balance it is to use the global threshold rather than local threshold for parameter *R*, because the global threshold considers mainly changing areas. However, the global threshold means that each image has a separate threshold, and it is unfair to compare with other algorithms which are without parameter tuning. Thus, local threshold has been adopted in our approach by choose *R* as follows:

(11) \begin{eqnarray*}R={C}_{coe}- \frac{{I}_{s\text{_}norm}}{2} \end{eqnarray*}

where *I*_*s*_*norm*_ is the normalized intensity of samples, and *C*_*coe*_ is correction coefficient. In the evaluation, we use *C*_*coe*_ = 0.51 in tramStation and fluidHighway and *C*_*coe*_ = 0.41 in streetCornerAtNight, winterStreet, busyBoulvard and bridgeEntry. *R* is typically bound within the range of [0.12, 0.4]. It means that with the decrease of color intensity, the local threshold *R* will be increased. Using Eq. (11) we can suppress the high sensitivity in dark areas.

**Table 1:** Results of all seven measures.

Videos	Recall	Specificity	fpr	fnr	pbc	Precision	F-measure
tramStation	0.7691	0.9955	0.0045	0.2308	0.9026	0.7737	0.7714
fluidHighway	0.6101	0.9893	0.0107	0.3899	1.7169	0.5000	0.5496
streetCornerAtNight	0.8904	0.9964	0.0036	0.1096	0.4117	0.5402	0.6724
winterStreet	0.6753	0.9906	0.0094	0.3247	1.8713	0.6874	0.6813
busyBoulvard	0.4177	0.9929	0.0071	0.5823	2.7403	0.6828	0.5183
bridgeEntry	0.6692	0.9964	0.0036	0.3308	0.8208	0.7299	0.6982

**Table 2:** F-measure comparison with other state-of-art algorithms^1^.

Algorithms	TS	FH	SC	WS	BB	BE
our approach	0.7714	**0.5496**	**0.6724**	**0.6813**	**0.5183**	**0.6982**
SUBsense	**0.7764**	0.3964	0.6036	0.4516	0.4251	0.3166
C-EFIC	0.7648	0.5480	0.6450	0.6348	0.4729	0.6183
EFIC	0.7621	0.5441	0.6705	0.6077	0.4182	0.5980
WeSamBE	0.7696	0.4432	0.6212	0.5211	0.4406	0.4101

**Notes.**

1 The results is based on used ground truth frames, there are:TS:tramStation(#1210 –#1310), FH:fluidHighway(#415 –#655),SC:streetCornerAtNight(#800 –#2999), WS:winterStreet(#900 –#1339), BB:busyBoulvard(#730 –#1744), BE:bridgeEntry(#1000 –#1749).

**Figure 6:**
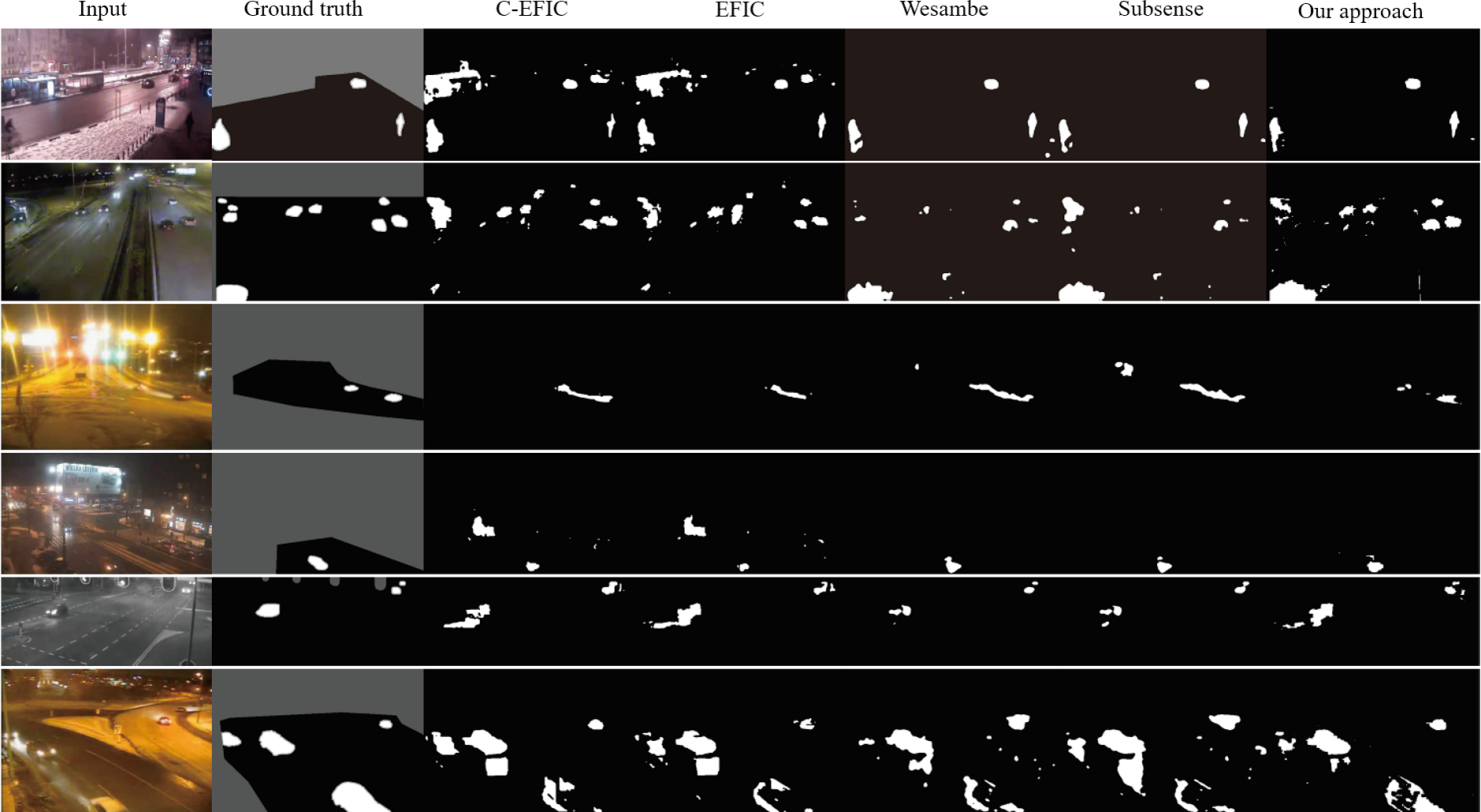
Result comparison with other algorithms. Input images from top to bottom are tramStation-#001131, fluidHighway-#000445, bridgeEntry-#001430, busyBoulvard-#001230, streetCornerAtNight-#002834, and winterStreet-#001225 (Wang et al., 2014).

### Evaluation

We evaluated our approach using the seven measures and their results are shown in Table 1. The F-measure plays an important role in the evaluation of overall performance. There are results of comparisons with other state-of-art algorithms as shown in Table 2 using series of video frames with ground-truth information. Our approach shows a competitive performance for night videos with dim objects. To illustrate the advantage of our approach, images representing typical conditions such as lighting and dim objects are shown in Fig. 6. For some frames, our approach does not perform as good as other approaches, which is mainly due to the lack in shadow detections. Our approach is implemented in python. This program has been optimized through *justintime*(*jit*) based on numba, running with Intel core i5 at 1.6GHz and the processing speed is three frames per second.

## Conclusion

We proposed a new framework by integrating and improving a number of feature extractors to allow background subtraction. Our framework can enhance foreground object representation and reduce the impact of light reflections for videos in the evening. Our results justifies our approach. In the future, the detection performance can be further improved using temporal information, such as the intensity difference of two near frames.

##  Supplemental Information

10.7717/peerj-cs.592/supp-1 Supplemental Information 1 Code

## Additional Information and Declarations

### Data Availability

The following information was supplied regarding data availability: The CDnet2014 (2014 Dataset) are available at http://changedetection.net/. Python scripts are available in the Supplemental File.
